# Enhancing Healthy Longevity: Scoping Review of Practices and Interventions

**DOI:** 10.2196/94085

**Published:** 2026-09-08

**Authors:** Angela Yee Man Leung, Karen Siu Lan Cheung, Kai-ling Ou, Ivy Yan Zhao, Min Qiu, Ka Ming Wan, Jed Montayre, Yaqi Huang

**Affiliations:** 1 WHO Collaborating Centre for Community Health Services, School of Nursing, The Hong Kong Polytechnic University, 5th Floor, Core G, Hung Hom, Kowloon, Hong Kong, China (Hong Kong), 852 27665587; 2 School of Nursing, The Hong Kong Polytechnic University, Hong Kong, China (Hong Kong); 3 University of British Columbia, Vancouver, BC, Canada; 4 JBI Hong Kong Centre of Evidence-based Healthcare Excellence, School of Nursing, The Hong Kong Polytechnic University, Hong Kong, China (Hong Kong)

**Keywords:** healthy longevity, strategies, practices, policies, community-based, review

## Abstract

**Background:**

Over the past 2 decades, concerns have arisen about the distinction between health span and lifespan, highlighting that longevity does not necessarily equate to good health, a concept often referred to as “healthy longevity.” While various strategies have been explored to promote healthy aging and achieve healthy longevity, it remains uncertain which practices are most effective.

**Objective:**

This scoping review identifies the existing and emerging practices and interventions that promote healthy longevity, identifies the key components of these practices and interventions, and considers how stakeholders contribute to these practices and interventions.

**Methods:**

A scoping review of the literature was conducted using Arksey and O’Malley’s 6-stage framework. The Joanna Briggs Institute Population-Concept-Context framework was used to define the eligibility criteria and select studies reporting practices or interventions aimed at achieving a long health span or promoting healthy longevity carried out in the community. Data were manually extracted by 2 independent reviewers to detail the characteristics of these practices and interventions and guide the data charting process and narrative synthesis. Six databases (PubMed, Web of Science, Embase, Scopus, CINAHL, and Google Scholar) were searched for academic papers published between January 2010 and February 2025.

**Results:**

A total of 21 studies met the inclusion criteria. Most studies (n=15, 71%) were published after 2020 and were predominantly conducted in high-income settings (n=17, 81%) across North America, Europe, and Asia. Four study types were identified: interventional (n=7, 33%), intervention development (n=4, 19%), association (n=5, 24%), and descriptive (n=5, 24%). Interventional and intervention development studies primarily described multicomponent programs targeting individual and social determinants of health and generally reported beneficial effects on physical, cognitive, and psychosocial outcomes. Association studies linked micro-, meso-, and macro-level factors (eg, nutrition, household expenditure, housing quality, health insurance, and public financing policy) to healthy longevity indicators. Descriptive studies highlighted themes across the micro and meso levels, including finance, physical activity, mental and spiritual health, digital literacy, independent living, safety, social support, and health care support.

**Conclusions:**

The identified community-based strategies, practices, and policies that extend health span represent the joint efforts of multiple stakeholders and disciplines. The implementation of these practices and policies is worthy of being supported. More studies in diverse socioeconomic contexts are needed.

## Introduction

### Background

The concept of health span has increasingly attracted considerable attention within communities as longevity rises; however, not all individuals experience good health in their later years. Health span is defined as the duration of life spent in good health, encompassing psychological well-being, functional independence, cognitive integrity, and the absence of overt disease and disability [1]. These conditions are generally perceived positively within communities, as older adults tend to use fewer resources, such as health care services, rehabilitation, and social care support. An extended health span is highly esteemed due to its association with overall well-being, enhanced quality of life, and increased levels of life satisfaction in late adulthood [1,2].

In the past 2 decades, concerns have emerged regarding the distinction between health span and lifespan, emphasizing that longevity does not inherently equate to health, often termed “healthy longevity.” Some researchers argue that the gap between lifespan and health span is widening [3]. Lifespan refers to the duration of an individual’s life from birth to death, focusing solely on the number of years lived, irrespective of health conditions. Although lifespan has increased, many individuals spend the latter decade of their lives contending with significant health challenges [3]. Consequently, these individuals experience what is termed a “long lifespan but short health span,” suffering the consequences of prolonged ill health that necessitates intensive care or rehabilitation. This disparity between health span and lifespan is increasingly recognized as a global phenomenon. Recent data indicate that the health span-lifespan gap has expanded among 183 World Health Organization (WHO) member states, averaging 9.6 years, posing significant challenges to healthy longevity worldwide [3]. Notably, women exhibit a larger health span-lifespan gap compared to men, primarily owing to a greater burden of noncommunicable diseases [3].

To narrow the health span-lifespan gap, governments have sought to promote healthy aging by mobilizing community members and resources to support individuals to lead healthy and independent lives. This initiative aims to address declines in intrinsic capacity and maximize functional ability through interactions with the environment. Key strategies for promoting healthy aging include encouraging regular exercise, maintaining a healthy diet, and increasing social interaction among older adults. The United Nations Decade of Healthy Aging (2021‐2030) seeks to shift the global focus from mere longevity to healthy longevity [4,5]. Investments in initiatives that support healthy aging are crucial for preventing health deterioration alongside increasing longevity, which can lead to functional dependency and disability. Ultimately, such efforts benefit society by reducing overall health care costs.

Joint efforts from the community to achieve healthy longevity (or a long health span) have been consistently advocated. Some community-based practices are culturally specific and initiated by Indigenous Peoples, rooted in their beliefs and local resources. While these practices may be descriptive and lack thorough efficacy analysis, they should nonetheless be valued and respected. Additionally, initiatives from nonhealth sectors, such as housing and finance, contribute important perspectives to the concept of healthy longevity. However, the most effective practices or interventions to support healthy longevity for community-dwelling older adults—enabling them to maintain health, safety, and independence while engaging in cherished activities—remain unclear. To date, no scoping review has been conducted to examine existing and emerging practices and interventions that promote healthy longevity. This study addresses this critical knowledge gap by identifying useful practices and interventions, thereby providing a theoretical foundation to guide healthy longevity initiatives.

### Objective

This scoping review aims to identify existing and emerging practices and interventions that promote healthy longevity, highlight the key components of these interventions, and describe the actions stakeholders should take for the development and implementation of these practices or interventions in the community.

## Methods

### Design

A scoping review was chosen instead of a systematic review because it is particularly suited to exploring emerging topics, such as practices that promote healthy longevity, for which conceptual and empirical clarity is still limited [6]. This approach provides a comprehensive overview of the existing literature on the subject, reviews evidence rapidly, and identifies knowledge gaps. The scoping review process followed the guidance of the 6-stage framework described by Arksey and O’Malley [7] and further refined by subsequent authors, including (1) identifying the research questions; (2) identifying relevant studies; (3) selecting studies; (4) charting the data; (5) collating, summarizing, and reporting the results; and (6) the consultation exercise. The review protocol was not preregistered. Ethical approval was not required for this review of the existing literature. The review was reported in accordance with the PRISMA-ScR (Preferred Reporting Items for Systematic Reviews and Meta-Analyses Extension for Scoping Reviews) checklist (Checklist 1). A retrospective protocol was registered on OSF [8].

### Scoping Review Questions

The specific review questions were as follows: (1) What existing or emerging community-based practices and interventions support healthy longevity? (2) What are the key components of these practices and interventions? (3) How do different stakeholders contribute to these practices and interventions? Framework Stage 1: Identifying the Research Questions.

### Inclusion and Exclusion Criteria

Eligibility criteria were defined according to the Joanna Briggs Institute Population-Concept-Context framework [9].

Population: The included studies focused on middle-aged and older populations (aged 50 years and older), irrespective of gender, socioeconomic status, or health conditions.

Concept: The scope of this review was informed by the conceptual distinction between lifespan and healthy longevity and by the WHO’s healthy aging framework [10]. Lifespan refers to the duration of an individual’s life from birth to death [11], whereas healthy longevity, health span, or healthy lifespan emphasizes the period of good health and performance [12]. WHO defines healthy aging as the process of developing and maintaining the functional ability that enables well-being in older age. This framework emphasizes home, community, and broader societal environments as key contexts that support older adults’ functional ability [10].

In line with these frameworks, this review focused on practices, programs, strategies, or interventions intended to enhance health span, healthy aging, healthy longevity, or healthy life expectancy in community or home settings. Studies focusing solely on biological lifespan extension, or antiaging pharmacological or medication treatments were excluded because they did not address the community-based and health span–oriented focus of this review. Similarly, studies targeting a single clinical risk or disease outcome without reference to broader healthy aging or health span–related aims were outside the scope of this review. Such studies were eligible only when they were explicitly framed as contributing to healthy aging, health span, healthy longevity, or healthy life expectancy or when relevant outcomes related to these concepts were reported.

In this review, practices were defined as activities adopted by older individuals or communities, including those shaped by cultural beliefs, values, or everyday routines. Interventions were defined as structured activities, programs, strategies, or initiatives developed based on empirical evidence, theories, or pilot tests in selected populations. Interventions that were under development or being evaluated in trials were also eligible.

Context: All geographic locations and countries across various income levels (low-, middle-, and high-income) were included. Consistent with the WHO healthy aging framework, this review focused solely on strategies, programs, and initiatives that took place in community or home settings. Practices in institutions, including hospitals, clinics, long-term care institutions, or residential care homes, were excluded because resources in these in settings differ from those everyday living environments.

All study designs (such as randomized controlled trials, quasi-experimental studies, cohort studies, observational studies, and qualitative research) were included to capture the breadth of the literature. Only studies published in English between January 2010 and February 2025 were considered for inclusion. These studies had to explicitly indicate that the aims or outcomes of the practices or interventions were related to improving health span, healthy lifespan, healthy longevity, or healthy aging, be published in peer-reviewed English-language journals, and be conducted in community settings. Animal studies and in vitro research were excluded. Framework Stage 2: Identifying Relevant Studies.

### Search Methods

A comprehensive search and review of the literature published between January 2010 and February 2025 were conducted using databases and platforms including PubMed, Web of Science, Embase, Scopus, CINAHL, and Google Scholar, and searched the reference lists of the included studies. Proximity operators were used in the search strategy (eg, longev* was used to search for longevity).

The search strategy was intentionally bounded by the WHO healthy aging framework and the distinction between lifespan and health span. The aim was to identify community- and home-based practices, programs, strategies, and interventions that support health span, healthy aging, or healthy longevity, rather than biological lifespan extension alone. The Boolean search combined 3 concept blocks: (1) healthy longevity–related terms referring to the review of the health span definition [12], (2) intervention or practice-related terms, and (3) settings. The core search string was: (healthspan OR healthsp* OR “healthy aging” OR “healthy aging” OR “healthy ag*” OR “healthy longevity OR “healthy longev*” OR “healthy life expectancy” OR “healthy life exp*“) AND (intervention* OR practice* OR program* OR program*) AND (community OR home) NOT (hospital OR clinic OR “institutional setting” OR “residential care homes” OR institutions), including MeSH and keywords. The term “longevity” was retained as a supplementary broad aging term to capture studies in which health span–related outcomes were described. Search strings were adapted to the syntax and field tags of each database. The search was further refined using relevant subject categories, such as Health Care Science Services, Health Policy Services, and Good Health and Wellbeing, wherever it was available in the database. Framework Stage 2: Identifying Relevant Studies.

### Search Selection and Outcome

Following the search, all identified articles were collated in EndNote [13] and uploaded into the online systematic review software Covidence [14]. Two authors independently screened titles and abstracts against the inclusion criteria, followed by full-text review. Any disagreements between the authors at each stage of the selection process were resolved through discussion. If consensus could not be reached, a third author reviewed the study to make a final decision. Studies that did not meet the Population-Concept-Context criteria were excluded. Framework Stage 3: Selecting Studies.

### Data Extraction and Abstraction

After selection, the included articles were extracted by 2 authors using a predesigned data extraction form in Microsoft Excel. Study characteristics included the following details: authors, year, country, income level, study design, age, other conditions, and sample size. Additional details were extracted based on the study type. For interventional studies, we extracted the following information: authors, year, study design, funding of the program, and strategies, practices, or policies related to healthy aging. For intervention development studies, we recorded authors, year, study design, framework, and strategies, practices, or policies. For association studies, we extracted authors, year, study design, outcomes, and significant or nonsignificant factors related to healthy longevity. Additionally, the key themes identified in descriptive studies were categorized into micro-, meso-, and macro-level framework. Any discrepancy between the 2 reviewers was resolved through discussion, or by an additional member of the project team. Formal quality appraisal of the included studies was not undertaken, consistent with common practice in scoping reviews. These data are presented in the *Results* section. Framework Stage 4: Charting the Data.

### Synthesis

The recurring themes identified in the reviewed studies focused on the key components of the practices (interventions), the individuals involved in their delivery, the strategies employed, and the outcomes observed following implementation. Next, we conducted a narrative synthesis of the charted data. Strategies, practices, policies, and associated factors identified were mapped onto micro-, meso-, and macro-level determinants of healthy longevity to summarize patterns across study types (Framework Stage 5: Collating, Summarizing, and Reporting the Results). Using the micro-, meso-, macro-level framework, we applied established definitions for each level: the micro level is about individuals and one-on-one interactions, the meso level is about the community level, and the macro level is the societal level, including policy [15]. The optional Framework Stage 6 (Consultation Exercise) was not undertaken in this review.

## Results

### Study Selection

**Figure 1.**
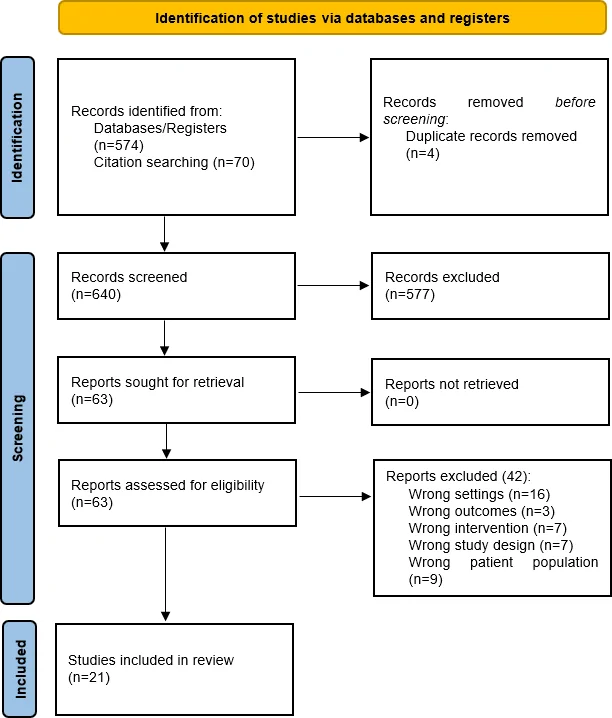
PRISMA-ScR (Preferred Reporting Items for Systematic Reviews and Meta-Analyses extension for Scoping Reviews) flowchart of the literature search strategy.

A total of 644 titles and abstracts were identified and screened to remove duplicates. After title screening, 504 articles were excluded. Sixty-three full-text articles were retrieved, of which 42 were excluded as they did not fulfill the eligibility criteria. Finally, 21 articles were included in this scoping review. Figure 1 details the results of the search, and the inclusion process is reported in full according to the PRISMA-ScR flow diagram [16].

### Study Characteristics

The studies included in this review were published between January 2010 and February 2025, with most published after 2020 (15/21, 71.4%). Most of the studies were conducted in high-income countries or economies (17/21, 81.0%), and the rest were in middle-income countries (4/21, 19.0%), with about one-third in North America (8/21, 38.1%), one-third in Europe (7/21, 33.3%), and Asia (6/21, 28.6%). Four of 21 (19.0%) studies were conducted in rural or low-income communities. Studies conducted in low-income communities mainly focused on basic preventive health risk practices using existing resources, while programs in other affluent settings implemented multidomain approaches. Among the 20 studies recruiting participants, 15 (75%) recruited participants or informants aged 60 years or older, and 10 (50%) studies had additional criteria for target participants: had chronic diseases, lived independently at their own home or lived alone, or lived in low-income areas.

In the review, we identified 4 approaches to the study of interventions for healthy aging: interventional studies (n=7), intervention development studies (n=4), association studies (n=5), and descriptive studies (n=5). First, interventional studies are those that used experimental designs to assess the impact of a specific intervention to improve healthy aging outcomes, including active aging, physical performance, cognitive performance, preventive health behaviors, survival rate, and health service utilization. Second, intervention development studies include those that reported the development of a specific intervention for healthy aging without empirical examination of its impact. Third, association studies used longitudinal studies examining factors associated with measures of healthy aging, such as mortality, disability-adjusted life years (DALYs), Activities of Daily Living/Instrumental Activities of Daily Living, and depression. Fourth, descriptive studies using qualitative methods illuminated participant perspectives and identified themes associated with interventions of healthy aging, such as safety environment, finances, and support systems. Each of these four approaches offers important insights into interventions affecting healthy aging. The sample sizes ranged from 30 to 462 for the 7 interventional studies, 18 to 112 for 4 intervention development studies, and were at least 3000 in 4 of the 5 association studies which had provided the information. For the descriptive studies, the sample sizes ranged from 19 to 47. Details of study characteristics are provided in Table 1.

**Table 1.** Study characteristics (n=21).

Authors, year	Country	Income	Study design	Age (y)	Other conditions	Sample size
Interventional studies						
Davodi et al [17], 2023	Iran	Middle	RCT^a^	≥60	—^b^	60
Newman et al [18], 2010	United States	High	Pre-and-post	≥65	Low-income high-risk community	389
Zgibor et al [19], 2017	United States	High	Cluster RCT	≥50	Arthritis and multimorbidity	462
Lee et al [20], 2022	Taiwan	High	Secondary data analysis of RCT	≥65	At least 3 chronic diseases	297
Moriyama et al [21], 2023	Japan	High	Quasi-experimental	≥65	Not receiving support/certification for long-term care	307
Carter et al [22], 2021	United States	High	Pre-and-post	≥55	Live in low-income housing	30
Lucifora and Villar [23], 2024	Italy	High	Quasi-experimental	≥65	Retirees of a private company	356
Intervention development studies						
Joymangul et al [24], 2024	Italy, Romania, and Portugal	High	Mixed methods observational	No info	—	112
Orellano-Colón et al [25], 2014	Puerto Rico	High	Mixed methods	>70	Older adults who lived alone	Older adults: 8, community key members: 4; researchers: 6
Gwyther et al [26], 2018	Greece, Italy, the Netherlands, Poland, Portugal, Spain, the United Kingdom	High	Mixed methods	N/A^c^	N/A	21 project partners
Ashikali et al [27], 2024	Switzerland	High	A participatory approach	≥65	—	36 (older adults: 4; HCPs^d^: 13; SCPs^e^: 9; community representatives: 10)
Association studies						
Nie et al [28], 2021	China	Middle	Longitudinal	≥60	—	8839
Wu and Grundy [29], 2025	United Kingdom	High	Longitudinal	≥65	—	4710
Zai [30], 2024	United States	High	Longitudinal	≥65	At risk of requiring long-term care	3000
Dragos et al [31], 2022	26 European countries	High	Longitudinal panel	No info	—	No information
McIntyre et al [32], 2021	United States	High	Longitudinal	≥18	—	Training: 2634; validation: 2505
Descriptive studies						
Ienca et al [33], 2021	Switzerland	High	Semistructured interviews	≥65	Lived independently in their own home	19
Solhi et al [34], 2022	Iran	Middle	Semistructured interview	45‐59	—	21
Puplampu et al [35], 2022	Canada	High	Semistructured interviews	≥65	—	19
Somrongthong et al [36], 2014	Thailand	Middle	Semistructured interviews with site observation	≥65	Rural community	47 (older adults: 16; family: 17; volunteers: 11; staff: 3
Bacsu et al [37], 2012	Canada	High	Ethnographic approach with interviews	≥65	Rural community	42

a RCT: randomized controlled trial.

b Not available.

c N/A: not applicable.

d HCP: health care professional.

e SCP: social care professional.

### Interventional and Intervention Development Studies

Seven studies described specific interventions to improve healthy aging, with various study designs, including RCTs or cluster RCTs (n=3), quasi-experiment with control group (n=2), and a predesign and postdesign (n=2) (Table 2). No study aimed at the macro level, 1 study targeted integration of care at meso level [22], 2 studies targeted lifestyle modifications at the micro level [20,21], and the remaining 4 studies examined multidomain interventions, with most components at the micro level, including physical exercise, nutrition, stress/depression management, and preventive health behaviors, and also one component (social contact/engagement) at the meso level [17-19,23]. Outcome measures were divided into two categories: (1) epidemiological outcomes such as active aging scores [17], survival rate [21], and disability-free life expectancy [23], and (2) physio-psychosocial outcomes: physiological measures [18], cognitive performance [20], social engagement [17,19,23], and mental health [23].

**Table 2.** Details and outcomes of the interventional studies (n=7)^a^.

Authors, year	Funding of the program	Strategies/practices/policies of healthy aging	Outcomes
Davodi et al [17], 2023	Research study	Micro Intervention: Nutrition Physical activity Responsibility for preventive behaviors Stress management Spiritual aspects Control: Routine care Meso Communications	(+) Total active-aging score increased (68.5±3.0 → 85.0±8.3, *P*<.001) (+) Significant improvements were observed in active-mind maintenance (9.0±2.0 → 13.0±4.0, *P*<.001), physical-functional activity (15.0±4.0 → 16.0±4.0, *P*=.03), social contact (11.5± 4.0 → 16.0±3.3, *P*<.001), productive engagement (13.5±6.0 → 19.0±6.0, *P*<.001), and social-institutional participation (6.0±1.0 → 9.0±4.0, *P*<.001). (=) No significant difference in agent attitude (13.7±1.60 →13.6±1.60, *P*=.96)
Newman et al [18], 2010	No funding	Micro The 10 keys: Blood pressure control Smoking cessation Immunization Cancer screening Regulating blood glucose Regulating cholesterol Physical activity Maintaining healthy bones, joints, and muscles Combating depression Meso Promoting social contact	(↑) Improvements were seen for the proportion of participants meeting goals for low-density lipoprotein cholesterol (+43%), blood pressure control in hypertensives (+17%), blood glucose control in diabetics (+50%), and colon cancer screening (+13%). Among those without prior vaccination, influenza vaccine increased by 25% and pneumonia vaccine by 20%
Zgibor et al [19], 2017	Research grant	Micro AEEP^b^ sessions Combining exercise Health education on chronic disease prevention and healthy aging The 10 keys Blood pressure control Smoking cessation Immunization Cancer screening Regulating blood glucose Regulating cholesterol Physical activity Maintaining healthy bones, joints, and muscles Combating depression Meso Promoting social contact	(=) Both groups improved, with no significant between-group differences in Short Physical Performance Battery (9.7±2.4 → 9.9 ± 2.3; *P*=.07) or Western Ontario and McMaster Universities Osteoarthritis Index (23.8±17.9 → 21.2±17.4; *P*=.92) at 6 months; (↑) Hypertension control increased from 60.1 → 76.7% (AEEP/10 keys) and 76.5 → 84.9% (AEEP alone), and diabetes control from 15.0 → 34.9% and 15.5 → 34.1%, respectively. These community-based programs showed similar improvements in preventive health, mobility, and arthritis outcomes
Lee et al [20], 2022	Research grant to the original RCT^c^	Micro Integrated geriatric care plus multidomain intervention Physical exercise Cognitive training Diet education Chronic condition management	Physio-Cognitive Decline Syndrome (+) Significant improvement in global cognition (Δ=1.1, 95% CI 0.4‐1.8; *P*=.003); domain-specific gains in concentration (0.3, 95% CI 0.1‐0.5; *P*=.01), language (0.2, 95% CI 0.1‐0.3; *P*=.006), abstraction (0.1, 95% CI 0.0‐0.3; *P*=.03), and orientation (0.2, 95% CI 0.0‐0.4; *P*=.01). (=) No significant change in visuospatial, naming, or delayed memory domains. ( + ) Improved physical-role-limitation domain of QoL^d^ (Δ=5.3, 95% CI 0.3‐10.4; *P*=.04). Cognitive impairment, no dementia (CIND) (–) Significant reduction in frailty (Δ=–0.3, 95% CI –0.5 to –0.1; *P*=.01). Mobility impairment, no disability (MIND) (–) Significant reduction in frailty (Δ=–0.3, 95% CI –0.4 to –0.1; *P*=.004)
Moriyama et al [21], 2023	Local Government	Micro Tai Chi Yuttari exercise performs in sitting or standing position	(+) Participation in Tai Chi Yuttari classes was associated with significantly longer survival (*χ*²=8.782, *P*=.003) and longer long-term care duration (*χ*²=5.354, *P*=.02) compared to nonparticipants; effect of survival duration is significant in men only (*χ*²=7.875, *P*=.005). Participation in Tai Chi Yuttari exercise might be effective in delaying death, especially in men, and new certification for long-term care
Carter et al [22], 2021	Research grant	Meso University unit partners with low-income housing to establish a clinic in the housing estate so that older people can easily access health services, forming an integrated network of care and promoting aging-in-place	After 1 year (=) Emergency visits and hospitalizations remained stable, but the reasons for emergency visits shifted toward acute issues. (↑) Follow-up adherence improved slightly, while missed appointments increased a bit. (+) Use of ancillary services (physiotherapy, occupational therapy, home care, nutritional counseling, behavioral health, etc) expanded significantly. (↑) Vaccination rates showed a modest improvement overall
Lucifora and Villar [23], 2024	Private company	Micro Personalized medical health plans Physical domain (physical activity courses and Tai Chi classes) Mental well-being domain (activity for boosting creativity and memory) A multidimensionality of the healthy aging intervention Meso Social domain (activities for improving social engagement)	(↑) Increased healthy lifestyle adoption by 23%‐25% and social engagement by 18%‐19% (↓) Reduced poor physical health by 3%‐4% and poor mental well-being by 16%‐17% (in ≥75 years) (↑) Extended disability-free life expectancy by ≈5 years (14 vs 9 years)

a (+)=significant increase/improvement (*P*<.05); (–)=significant reduction (*P*<.05); (↑)/(↓)=nonsignificant trend toward increase/reduction (*P*≥.05); (=)=no significant change/difference.

b AEEP: Arthritis Foundation Exercise Program.

c RCT: randomized controlled trial.

d QoL: quality of life.

Six out of 7 studies involved physical activity in interventions [17-21,23] for healthy aging program. One quasi-experimental study found that Tai Chi participation could significantly increase survival duration especially in male participants [21]. This suggests that demographic characteristics, such as sex, should be explicitly considered when designing and targeting healthy aging interventions. The only cluster RCT in this review showed that preventive interventions addressing hypertension and diabetes control, lifestyle modification, and social contacts could be executed by community health workers, resulting in improvement in mobility, arthritis outcomes, blood pressure, and blood glucose [19]. Another pre-post intervention study using similar preventive strategies for chronic condition management, delivered by local medical staff and community health counselors, also demonstrated improvements in goal attainment for low-density lipoprotein cholesterol, blood pressure control, blood glucose control, and colon cancer screening [18]. These 2 studies illustrate the manpower needed to support health span or healthy aging in communities.

Another illustrative intervention for promoting aging-in-place and minimizing health disparity is the pilot cohort study in America, in which a university unit partnered with a housing operator to set up a primary care clinic in a low-income housing development. One-year follow-up showed that the use of ancillary services (such as physiotherapy, nutritional counseling, etc) increased significantly [22]. This indicates the importance of the removal of transportation barriers to accessing health services in the community.

Three RCT studies designed and implemented a multidomain health promotion education program incorporating components on nutrition [17,20], physical activity [17,20,23], smoking and tobacco control [17], mental well-being [17,23], social communication [17,23], spiritual activities [17], and chronic condition management [20]. These interventions significantly improved active and healthy lifestyles, physical health, and social participation [17,23], reduced physio-cognitive syndrome as well as cognitive and mobility impairment [20], and extended disability-free life expectancy [23]. These findings indicate that, beyond the macro-level strategies, active and healthy aging can be effectively promoted through multidimensional interventions at the micro- and meso-levels, underscoring the need for person- and community-level programs in routine practice. Among these interventions, 57% (4/7) of interventions demonstrated significant improvements in health outcomes. Improvements were effective when they incorporated multidimensional supports, involved participants with preserved functional capacity, and were delivered in settings backed by local community infrastructure.

Four studies reported the development of interventions designed to promote healthy aging (Table 3). All of them were multicomponent, with 1 study covering only components at the micro level (cognition, nutrition, depression, and physical performance) [27], and the other 3 studies intending to address components at the micro- and meso-levels [24-26]. One study emphasized the combination of in-person visits with technology in preparing personalized intervention for physical activity, self-management, social engagement, and digital literacy [24]. Another study focused on a culturally appropriate intervention to promote physical and mental health and spiritual issues at the micro level and social engagement and safety at the meso level [25]. Three studies developed their interventions based on a conceptual framework [24,25,27].

**Table 3.** Intervention development studies (n=4).

Authors, year	Framework	Strategies/practices/policies
Joymangul et al [24], 2024	Active Ageing and Personalised (AGAPE)	Micro A supervised learning algorithm combines in-person and technologically personalized intervention on 4 aspects: Physical activity Digital literacy Self-management Meso Social engagement
Orellano-Colón et al [25], 2014	Person Environment Occupational Performance Model	Meso A culturally appropriate intervention in language, facilitator, metaphors, and content on 6 aspects: Activity, Health, and Active Aging (Impact of activity on health, Impact of aging on activities routines) Maintaining Physical and Mental Wellbeing through Occupations (Keeping mentally active, Keeping physically active, Nutrition) Social Relationships (Building social support through activity participation, Effective communication and social interactions, Coping with loneliness) Home and Community Safety (Protection against crimes at home and the community, Fall hazards and home safety, Activity adaptative, assistive devices and home modifications for safety) Participation in the Home and Community (Leisure and voluntary activities and health, Transportation use, Finance management, Resources in the community for participation in activities) Personal enablers of occupational participants (Role of personal attributes in daily participants, Spirituality as an enabler for occupational participants)
Gwyther et al [26], 2018	—^a^	Micro Physical activity Cognitive function Nutrition Psychology support Meso Intergeneration activities Social care
Ashikali et al [27], 2024	WHO’s^b^ ICOPE^c^ approach	Micro Identify SMART^d^ goals based on personal priority regarding: Cognitive decline Limited mobility Hearing loss Malnutrition Vision impairment Depressive symptoms

a Not applicable.

b WHO: World Health Organization.

c ICOPE: Integrated Care for Older People.

d SMART: Specific, Measurable, Attainable, Realistic, and Time-bound.

### Association Studies

We found 5 studies that reported associations of important variables with various measures of healthy aging [28-32]. In these studies, measures were used to conceptualize healthy aging, such as Chinese Healthy Aging Index, Activities of Daily Living or Instrumental Activities of Daily Living, depression, DALYs, and biological age. These studies included 5 variables at the micro level, 2 at the meso level, and 5 at the macro level (Table 4). At the micro level, 4 out of the 5 variables were statistically and significantly associated with measures of healthy aging. Two variables, household expenditure and nutrition, were positively related to measures of healthy aging [28,32]. One study showed alcohol consumption and overwork were positively correlated with DALY, whereas eating habits showed nonsignificance [31]. People with more limitations in Activities of Daily Living were more likely to acquire housing adaptations at Meso level [29]. At the macro level, low housing quality, such as housing poverty, was associated with decreased healthy aging [28], while health insurance coverage and public financing policies were negatively associated with unhealthy ageing [30,31].

**Table 4.** Association studies (n=5).

Authors, year	Outcomes	Factors associated with the outcome
Nie et al [28], 2021	CHAI^a^ (Unhealthy Aging)	Micro Significant (+) Household expenditure Macro Significant (+) Housing poverty
Wu and Grundy [29], 2025	Slower Activities of Daily Living/Instrumental Activities of Daily Living development	Meso Significant (+) Acquiring housing adaptation Nonsignificant Sex
Zai [30], 2024	Poor health; Mobility limitation; Instrumental Activities of Daily Living limitation; Depression	Macro Significant (−) Medical Aging Waiver program expenditure per capita
Dragos et al [31], 2022	Disability-adjusted life years (DALY)	Micro Significant (+) Alcohol consumption (+) Overwork Nonsignificant Eating habits Macro Significant (−) Voluntary health insurance (−) Quality of institutions (−) Public financing (voluntary health insurance)
McIntyre et al [32], 2021	Biological age deceleration	Micro Significant (+) Nutritional components: fiber, magnesium, and vitamin E intake

a CHAI: Chinese Healthy Aging Index.

### Themes From Descriptive Studies

Five studies were descriptive studies that explored participants’ perceptions of healthy aging [33-37]. A total of 6 themes at the micro level and 5 at the meso level were identified, but none at the macro level, and they were similar to the interventional components in the interventional, intervention development, and association studies. The 6 micro-level themes included finance, nutrition, physical activity, mental and psychological management, spiritual aspect, and digital literacy, whereas the 5 meso-level themes were independent living/housing, safety, communication, support systems, and health care support (Table 5).

**Figure 2.**
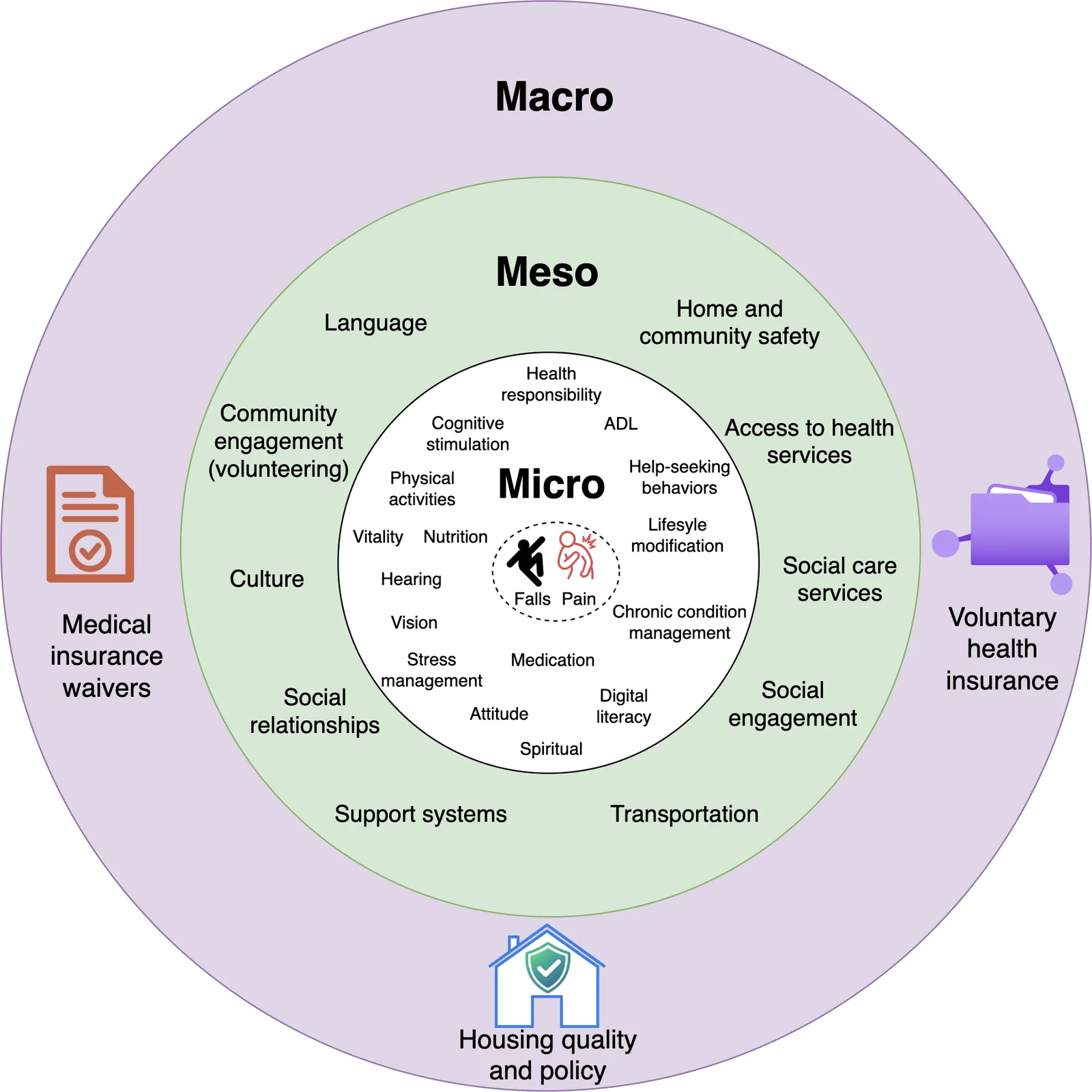
Strategies, practices, and policies of healthy aging at the micro-, meso-, and macro-levels. ADL: activities of daily living.

Figure 2 summarizes the key findings of this review.

**Table 5.** Descriptive studies (n=5).

Micro-, meso-, macro-level framework	Themes identified from the descriptive studies
Micro	Finance [34,37] Nutrition [34] Physical activity [34] Mental and psychological management [34,35] Spiritual aspect [34] Digital literacy [33,35]
Meso	Housing and community environment for independent living [33,37] Safety [33,35,36] Communication [34,35] Support system [35,37] Health care support [36,37]

## Discussion

### Principal Findings

This scoping review mapped the existing evidence on practices and interventions for healthy aging and health lifespan between 2010 and 2025. We identified 21 studies, of which 15 (71%) were published after 2020 and all studies were conducted in high- and middle-income countries, with 17 (81%) in high-income communities. Four study designs were evident: interventional studies, intervention development studies, association studies, and descriptive studies. Among these practices and interventions, 13 (62%) focused on micro-level factors (eg, physical activity, nutrition, mental and psychological management, spirituality), 11 (52%) focused on meso-level factors (eg, social engagement, support systems, housing and health care support). Very few practices (n=2, 9.5%) addressed macro-level determinants such as public financing or policy. Overall, the findings suggest a growing interest in multidomain programs that integrate micro- and meso-level components, but with important gaps in evidence from low-resource settings and in macro-level strategies.

### Micro‑ and Meso‑Level Factors

Multidomain interventions, including physical exercise, nutrition, emotional management, lifestyle modification, and social engagement, were the most frequently reported interventions that bring benefits to older people to extend health span. This finding is consistent with previous research, showing that interventions can improve physical performance, cognitive function, nutritional status, mood, cardiovascular risk profiles, and overall quality of life at-risk of functional decline [38-40]. From a mechanistic perspective, the rationale for multidomain interventions is also plausible. Aging is driven by multiple hallmarks, resulting from both endogenous and exogenous stress [41]. Multidomain interventions may act simultaneously on several of these hallmarks [42]. For example, physical activity and nutrition can enhance metabolic regulation, cognitive and social engagement can reduce neuroinflammation, and psychological support can improve stress resilience. Although the included studies rarely measured biological mechanisms directly, this framework provides an explanation for why interventions targeting multiple domains can produce broader benefits of healthy longevity [41].

Nevertheless, multidomain interventions should not be implemented as fixed, one-size-fits-all packages. Their effects may vary according to population characteristics, delivery modes, study settings, intervention intensity, and outcome measures [43,44]. Therefore, how practices and interventions are delivered appears to be important. In this review, evidence across interventional, developmental, associative, and descriptive studies further highlighted the importance of social environmental factors at the meso level, including culture [25], home and community safety [25], housing conditions [29], support systems [35], social care services [26], access to health services [22], and social engagement, etc. Culturally adapted interventions can improve adherence because they are more likely to fit older adults’ language, food habits, family structure, religious beliefs, and social expectations [45,46]. Similarly, housing quality and neighborhood safety are important for aging in place. Large-scale analyses have shown that environmental factors account for a substantially greater proportion of variation in mortality risk than genetic predisposition (around 17% vs <2%) [47]. Poor housing may increase exposure and exert particularly strong effects on diseases of the lung, heart, and liver [47], while unsafe homes and communities may increase falls, disability, and hospitalization [48]. In contrast, safe and accessible neighborhoods may support social engagement, mental health, and quality of life [49,50]. Moreover, community-based interventions create opportunities for social interaction to mitigate loneliness and social isolation and thereby influence both psychosocial and biological pathways [51,52]. Given the heterogeneity in intervention components observed in this review, more studies can examine which combinations and dosages of multidomain interventions are most effective for specific populations and delivery conditions.

Our review found that interventions delivered by community health workers improved preventive health behaviors and increased the use of health services [18,22]. These findings indicate that healthy aging interventions also need to be supported by accessible community services and local workforce capacity. Considering both micro- and meso-level factors, future interventions should adopt person-centered designs that match components to older adults’ intrinsic capacity, goals, preferences, and social environments, etc. Personalized approaches, such as the ICOPE (Integrated Care for Older People) framework [53], may support the codevelopment of tailored care plans and help translate multidomain interventions into routine practice.

### Macro‑Level Factors

At the macro level, long-term care financing policy and health system financing policy, such as medical insurance waiver programs and voluntary health insurance, influence healthy lifespans by reducing financial barriers and improving access to care. Similar to previous multicountry studies, this review found that universal health coverage was positively associated with both life expectancy at birth and healthy life expectancy [54]. Evidence from China further showed that health insurance was associated with higher end-of-life medical expenditure among older adults, suggesting improved access to medical services [55]. However, because all the involved studies were from high-income countries, unequal insurance benefits across different income settings need to be further explored. In addition, none of the interventional and qualitative studies targeted macro-level practices. Future research should move beyond micro- and meso-level approaches and examine how policy, public hospital authority intervention, financing systems, support for low-income housing, and equitable access to services can support healthy longevity. This gap is also important from a life-course perspective. Although most included studies focused on later-life practices and interventions, protection policies are needed to ensure that health-promoting environments and resources are available throughout the life course [56]. Early macro-level interventions aimed at reducing socioeconomic disparities helped narrow health gaps in older age [57]. This is particularly important for older adults living in low-income, rural, minority, or otherwise underserved communities.

### Evidence Gaps in Low‑Resource Settings

All studies included in this review were from high- or middle-income countries. While evidence from low-income countries was limited. This limits our understanding of how healthy longevity interventions operate in settings with fewer financial, technological, and service resources. Studies conducted in low-income communities in this review appeared to focus mainly on basic health risk prevention and the use of locally available resources, whereas programs in more affluent settings were more likely to adopt multidomain, service-integrated, or technology-enhanced approaches. This disparity is worthy of attention. Limited financial, health workforce, and technological capacity constrains the feasibility of multidomain interventions in resource-poor settings, which places populations at risk of widening inequalities in access to healthy aging support [37]. Therefore, there is a genuine need for developing and testing low-cost, high-reach strategies for healthy aging, such as community-based, peer-led, and integrated primary care to support older people’s functional capacities. Sustained strategies that promote health span require integrated care and collaboration across sectors, including health care professionals, social workers, community organizations and policymakers, particularly for older adults with lower socioeconomic status and multimorbidity. As discussed above, ICOPE is a potential approach [53] to enhance access to care and support intrinsic capacity, but its implementation depends on cross-sector collaboration. Pilot programs in several regions have demonstrated feasibility and acceptability [58]. Future research should draw on implementation science and mixed methods approaches to systematically document the facilitators and barriers to intersectoral collaboration, assess readiness and feasibility of related models in diverse health care systems [59]. These steps are crucial to move from small-scale trials toward sustainable system-level practice.

### Future Directions

**Figure 3.**
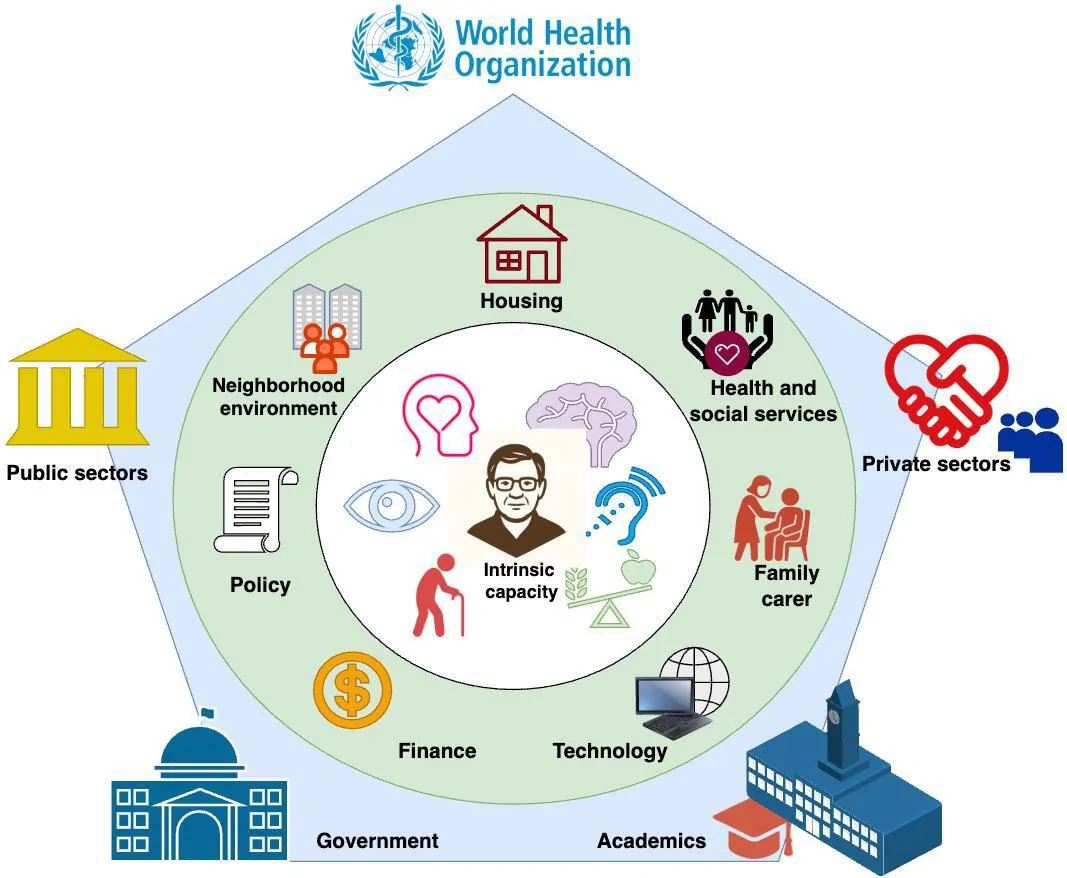
Conceptual framework summarizing multilevel practices supporting healthy aging and healthy lifespan.

Based on the findings of this review, we propose a conceptual framework that summarizes how healthy aging and healthy lifespan may be supported through multilevel practices (Figure 3). At the center of the framework is intrinsic capacity, which is shaped by individual-level behaviors and multidomain interventions, meso-level environments and services, and macro-level policies and financing systems. The framework also highlights the importance of cross-sector collaboration among government, public and private sectors, academia, communities, and international organizations.

### Limitations

This scoping review had a few limitations. First, we focused on studies that explicitly framed their practices or interventions around health span, healthy aging, lifespan, or longevity. So, treatment-oriented interventions such as fall prevention, frailty intervention, or multimorbidity management may have been excluded if they did not explicitly use health span or related terminology, even though such interventions may contribute to healthy longevity. Therefore, the findings should be interpreted as mapping explicitly health span or healthy aging-oriented practices rather than providing a comprehensive review of all interventions that may promote healthy longevity. Second, the components of some initiatives were not well-documented, which may lead us to overlook key elements necessary for extending health span. Third, most included studies (17/21, 81.0%) were conducted in high-income settings, which may limit generalizability. The results should therefore be interpreted with caution, particularly when applying these findings to low-resource contexts. Fourth, the terminological fragmentation observed across studies, where terms such as healthy aging, active aging, successful aging, and healthy life expectancy were used interchangeably. This inconsistent concept may hinder evidence synthesis. Therefore, establishing more standardized terminology would enhance comparability and translation of evidence into practice. Finally, healthy longevity practices or programs that are not documented in peer-reviewed literature were not included. Expanding this review to include gray literature could provide a broader understanding of effective practices in healthy longevity, including those executed by underrepresented populations.

### Conclusion

This scoping review identified key components of community-based strategies, practices, and policies aimed at extending health span and promoting healthy longevity, which warrant further testing and implementation. Overall, the existing evidence suggests that promoting healthy longevity in the community requires cooperation among multiple stakeholders, as well as the provision of integrated, multilevel services or support for older people. Contributions from different sectors and disciplines across all levels are equally important.

Existing interventions remain concentrated in high-income settings and at the micro level, particularly through the use of technology and multidomain integrated services to support older people in living independently and healthily within their communities. In contrast, strategies in low-income contexts largely focus on basic health risk prevention and the mobilization of local resources, such as volunteers, family members, and neighbors to support older people.

Macro-level strategies are especially critical, as policies at this level can promote health equity and reduce socioeconomic inequalities. There is an urgent need to further investigate and develop meso- and macro-level interventions across diverse socioeconomic contexts.

## Supplementary material

10.2196/94085 Checklist 1 PRISMA-ScR checklist.

## Abbreviations

DALY: disability-adjusted life year
ICOPE: Integrated Care for Older People
PRISMA-ScR: Preferred Reporting Items for Systematic Reviews and Meta-Analyses extension for Scoping Reviews
RCT: randomized controlled trial
WHO: World Health Organization
